# The Effect of Cosmetic Treatment and Gel Laser Therapy on the Improvement of Comedogenic Skin Type

**DOI:** 10.3390/gels9050370

**Published:** 2023-05-01

**Authors:** Jana Pavlačková, Hana Pecháčková, Pavlína Egner, Pavel Mokrejš, Robert Gál, Magda Janalíková

**Affiliations:** 1 Department of Fat, Surfactant and Cosmetics Technology, Faculty of Technology, Tomas Bata University in Zlín, Vavrečkova 275, 760 01 Zlín, Czech Republic; pavlackova@utb.cz (J.P.); pechackovahana@seznam.cz (H.P.); egner@utb.cz (P.E.); 2 Department of Polymer Engineering, Faculty of Technology, Tomas Bata University in Zlín, Vavrečkova 275, 760 01 Zlín, Czech Republic; 3 Department of Food Technology, Faculty of Technology, Tomas Bata University in Zlín, Vavrečkova 275, 760 01 Zlín, Czech Republic; gal@utb.cz; 4 Department of Environmental Protection Engineering, Faculty of Technology, Tomas Bata University in Zlín, Vavrečkova 275, 760 01 Zlín, Czech Republic; mjanalikova@utb.cz

**Keywords:** skin, comedone, sebum, cosmetics, gel, laser therapy, *Cutibacterium acnes*, skin diagnosis, porphyrin

## Abstract

Comedogenic skin care receives little attention compared to the care or treatment of more serious acne manifestations. Traditional therapies may have limited success with potential side effects. Cosmetic care supported by the effect of a biostimulating laser may offer a desirable alternative. The aim of the study was to evaluate the biological effectiveness of combined cosmetic treatment with lasotherapy on comedogenic skin type using noninvasive bioengineering methods. Twelve volunteers with comedogenic skin type underwent a 28-week application of Lasocare Basic 645^®^ cosmetic gel containing *Lactoperoxidase* and *Lactoferrin* in combination with laser therapy (Lasocare^®^ method). The effect of treatment on skin condition was monitored using noninvasive diagnostic methods. The parameters were the amount of sebum, the pore count, the ultraviolet-induced red fluorescence assessment of comedonic lesions (percentage of the area and quantification of orange-red spots), hydration, transepidermal water loss, and pH. A statistically significant decrease in sebum production was observed on the skin of the treated volunteers, as well as a decrease in porphyrins, indicating the presence of *Cutibacterium acnes* populating comedones and causing enlarged pores. The balance of epidermal water in the skin was regulated adjusting the acidity of the skin coat in individual zones, which decreased the presence of *Cutibacterium acnes*. Cosmetic treatment in combination with the Lasocare^®^ method successfully improved the condition of comedogenic skin. In addition to transient erythema, there were no other adverse effects. The chosen procedure appears to be a suitable and safe alternative to traditional treatment procedures known from dermatological practice.

## 1. Introduction

The condition of the skin and its healthy appearance are important indicators that tell not only its overall state but also the functioning of the organism as a whole. It is a complex reflection of the psychosocial influence on people’s personality, their inclusion, and communication in society [1,2,3]. The set lifestyle involves the excessive use of various forms of cosmetic products containing various active substances. Manufacturers of approved cosmetic products declare not only their effectiveness but also their safety. The consumer expects from such products both an improvement in the appearance of the skin and support of its physiological functions. However, it is the consumer for whose skin an inappropriately chosen cosmetic product can become fatal. Ignorance of the properties of raw materials in products can be a risk factor for skin. Therefore, the right choice of cosmetic product plays a key role for specific skin type.

In cosmetic practice, there are increasingly more cases in which individuals with a hypersensitive skin type make an incorrect choice of a cosmetic product containing comedogenic ingredients [4,5,6] or in which long-term treatment of the skin with disinfectants may promote the formation of microcomedones or comedones that turn into inflammatory acne lesions of varying severity. Cosmetic products contain many substances that, when in contact with the physicochemical and metabolically active environment of the skin surface, can affect the state of cell membranes, the mutual cohesion of corneal cells, and membrane systems in intercellular spaces. When this barrier is disrupted, as a site of possible penetration of comedogenic substances, the condition can also worsen in non-oily skin types. Therefore, cosmetic products designed for skin with a tendency to develop primary manifestations of acne should not be greasy and should have a high concentration of surface-active substances. They should be noncomedogenic, nonallergenic, and contain photoprotectors and antioxidants. Suitable vehicles are hydrophilic matrices with a higher water content or hydrogels [7]. 

Hydrogels based on polysaccharides (mostly starch and xanthan gum) are prepared from water-soluble base materials by mixing them following a technological procedure consisting of heating the mixture to a certain temperature and then cooling it. The composition of the gel depends on the intended use, according to which the addition of other active ingredients is selected [8]. The properties of gel matrices can be modified by chemical crosslinking, physical crosslinking, ultraviolet (UV) crosslinking or polymer grafting. Hydrogels with different composition, size, shape, and morphology can be prepared by controlled polymerization [9]. The gel formulation characterization methods include physical characterization (pH, rheology, appearance, swelling behavior, and texture) and chemical characterization (biological tests, microbial and sensory assessment) [8,10]. Gel matrices rapidly transform into liquid consistency on contact with the skin surface, dry quickly, and form a film that leaves no stains or greasy texture. The active substances from the gel formulation resorb more quickly than, for example, those from an emulsion matrix, taking into account the physicochemical properties of the incorporated substance. Gel formulations are suitable for application in oily areas such as comedogenic-affected areas on the face. The user comfort of topical application is influenced by the composition of the gel matrix raw materials, in which the gel-forming substances are present, which determine its viscosity, flow properties, retention in the skin and penetration of the active substances [11]. 

Such a vehicle should not cause abnormal follicular keratinization (microcomedone formation) and clog the pilosebaceous unit glands [12]. Furthermore, it should not contribute to the development of acne conditions, regardless of the skin type and age [6]. The distribution of acne lesions is highly individually heterogeneous. Lesions may occur in an oily area in some people and in a dry area in others [13]. Acne-like inflammation of the skin caused by various factors that promote abnormal keratinization and desquamation of the follicular epithelium is called comedogenicity [5,14,15]. Initially, invisible lesions—microcomedones—may develop into clinically obvious comedones, either open or closed, within two months. Due to accumulated sebum, microcomedones and comedones are further colonized by bacteria, mainly *Cutibacterium acnes* (*C*. *acnes*), which promote the formation of inflamed acne lesions—papules and pustules. *C*. *acnes* bacteria produce proinflammatory mediators, free fatty acids, and porphyrins, that are responsible for the appearance of the inflamed acne lesions [12,16,17]. Porphyrins are native fluorophores that are strongly fluorescent. The intensity of follicular fluorescence and the extent of facial involvement are proportional to the population density of *C. acnes* and the content of porphyrins on the surface of the skin. Orange-red fluorescence indicates the presence of *C. acnes* bacteria in clinically unmanifested follicular impactions and microcomedones, including evident lesions—comedones, papules and pustules. Therefore, porphyrin production represents the basis for the establishment of the treatment of such affected areas. Intervention early after acne breakthrough significantly reduces the risk of significant scarring and therefore requires a multifactorial approach that requires different care regimens [18]. The ability to detect invisible lesions at an early stage and visualize advanced small acne lesions is useful for verifying the effectiveness of cosmetic and dermatological applications. For both types of applications, their safety and efficiency are crucial. The cosmetic industry has comedogenic prediction models for individual ingredients and final formulations [19] that should serve as a guide; however, not all ingredients may be harmful to certain types of skin. Highly comedogenic or moderately comedogenic ingredients are recommended to be completely avoided by people with hypersensitive skin or by people with a history of acne. When assessing the comedogenicity of the product, it is necessary to take into account the amount of comedogenic components in the product, the presence of other comedogenic components, whether the product will be of the rinse-off or leave-on type and on which part of the body the product will be applied. In addition to topical applications for the common treatment of mild to moderate inflammatory manifestations of *Acne vulgaris*, laser therapy can also significantly minimize comedogenic lesions [20]. A combination of both procedures, i.e., the application of noncomedogenic preparations and laser therapy, has proven to be very effective in radically reducing the number of *C. acnes* and thus minimizing comedogenic lesions [21,22,23,24]. In contrast to traditional treatment methods, including topical or oral antibiotics, retinoids, or keratolytics, an alternative method of treating comedogenic skin with gentle topical preparations and optical cosmetic skin treatment is proving to be a promising and less risky method without side effects [25,26,27].

The purpose of the study was to determine the effect multifactorial care consisting of topical application of a cosmetic gel containing *Lactoperoxidase* and *Lactoferrin* and the optical effect of the biostimulation laser using the Lasocare^®^ method on reducing the number of lesions in comedogenic skin types and minimizing their development in more severe acne stages, reducing the risk of scarring and minimizing the psychosocial impact. Hypothesis: The combined effect of a cosmetic gel with photosensitive enzymatic complexes of *Lactoferrin* and *Lactoperoxidase* together with the use of laser light will have a positive effect in precisely targeted oxygenation of cellular structures in the depth of the skin pores and its effective cleansing. 

## 2. Results 

### 2.1. Questionnaire Survey 

A questionnaire survey conducted before the experiment showed that two female volunteers had a genetic predisposition to acne from their parents. Ten volunteers had already observed the beginnings of acne symptoms during adolescence, and in two volunteers, the symptoms did not become apparent until adulthood. As another of the subjectively perceived factors contributing to the deterioration of the skin condition, volunteers mentioned staying in the sun and dehydration of the skin, although dehydration was not confirmed by corneometric results; see Section 2.3.2. During the experiment, none of the participants visited a dermatologist or received any other cosmetic treatment. The volunteers also stated that they were not taking any topical or oral medications that could affect the course of the experiment. After a series of treatments, the volunteers completed a questionnaire. Ten of them positively evaluated the therapy; only two volunteers did not notice any changes in their skin according to their subjective judgment.

### 2.2. Visual Skin Assessment

The following comedogenic severity distribution of the volunteers before and after the lasotherapy method was determined according to the classification scale used; see Table 1. Before starting the experiment, the skin of seven volunteers was assessed as problematic with comedones, papules, and pustules in the T + U zone, in four volunteers only in the T-zone, and in one only in the U-zone without the presence of pustules. After the end of the experiment, comedones and papules were evident in the T + U zone only in two volunteers and in the T-zone in nine volunteers with a score of 1 to 2, and in one of the volunteers, pustules were also observed. 

### 2.3. Skin Properties Evaluated by Noninvasive Bioengineering Methods

The following subsections show the results of the objective characterization of the skin condition after baseline diagnostics and after 4, 8 and 28 weeks of combined therapy application.

#### 2.3.1. Sebum Content

**Figure 1:**
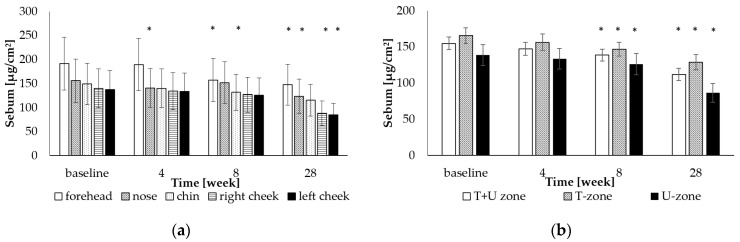
Comparison of the mean ± SD facial sebum secretion in: (**a**) forehead, nose, chin, left cheek, right cheek; (**b**) T + U zone, T-zone, U-zone; * indicates statistically significant difference from baseline (*p* < 0.05).

The sebum taken from individual zones on the face of the volunteers is compared in Figure 1. The monitored level of sebum from 138 to 191 μg/cm^2^ before the start of the experiment indicates a normal skin type, despite the visually noticeable comedoneness of the skin according to Table 1. The forehead, nose, and chin area (T-zone) had higher levels of sebum, where the sum of the values resulted in a level of 166 μg/cm^2^. A statistically significant decrease in sebum compared to the initial state of the skin was observed after 8 and 28 weeks (end of therapy). The total decrease in sebum in the T-zone was approximately 22%, and in the U-zone up to approximately 38%, compared to the initial values (*p* < 0.05).

#### 2.3.2. Facial Hydration, Transepidermal Water Loss and pH

**Figure 2:**
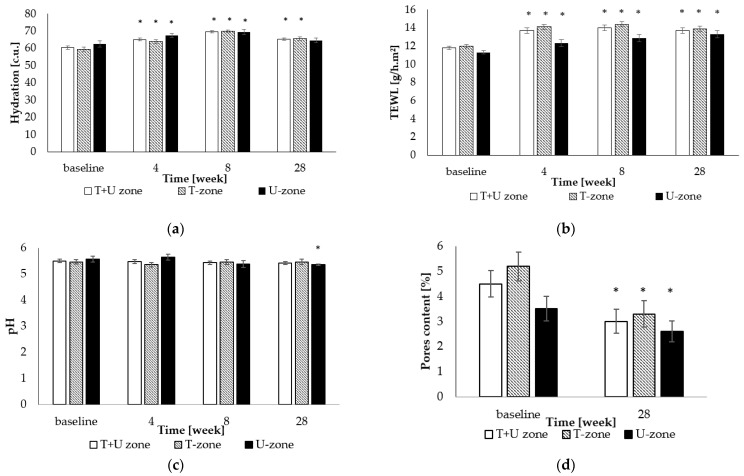
Comparison of mean ± SD: (**a**) facial hydration, (**b**) facial TEWL, (**c**) facial pH; (**d**) facial pores content; * indicates a statistically significant difference from baseline (*p* < 0.05).

The corneally monitored skin of the volunteers already showed sufficient hydration at the beginning of the experiment, an average of 60.4 ± 1.1 c.u., for both zones. With skin treatment, the hydration values compared to baseline (*p* < 0.05) fluctuated significantly over time, both on the forehead as well as on the chin and cheeks of the volunteers. More balanced hydrated skin of the entire face was achieved after 8 weeks of cosmetic treatment combined with the effect of biostimulation laser and home care. The recorder value was 69.6 ± 0.7 c.u., about 13% more than at baseline, as shown in Figure 2a. Another monitored parameter that can be used to assess the integrity of the *stratum corneum* as an indirect indicator of the functionality of the skin barrier [28] was transepidermal water loss (TEWL); Figure 2b. The average amount of evaporated epidermal water (11.8 ± 0.2 g/h.m^2^) at the beginning of the experiment indicated a functioning corneal layer. The same was true for the TEWL value found in the subsequent course of the experiment, despite its slight increase. Due to the set of cosmetic care and lasotherapy, epidermal water loss increased significantly in the observed periods from 1.9 to 2.2 g/h.m^2^ (*p* < 0.05), but within the range of values corresponding to the healthy functioning of the skin barrier. From the evaluation of the acidity of the skin coat, there was a significant difference in the U-zone between the value at the beginning of the experiment (pH 5.57 ± 0.11) and at the end (pH 5.35 ± 0.03). From the pH data obtained (Figure 2c), a decrease in these values is evident, which corresponds to a decrease in the inflammatory skin lesions of the volunteers. These demonstrated changes in pH can be attributed to changes in sebum levels.

#### 2.3.3. Facial Pores Content

**Figure 3:**
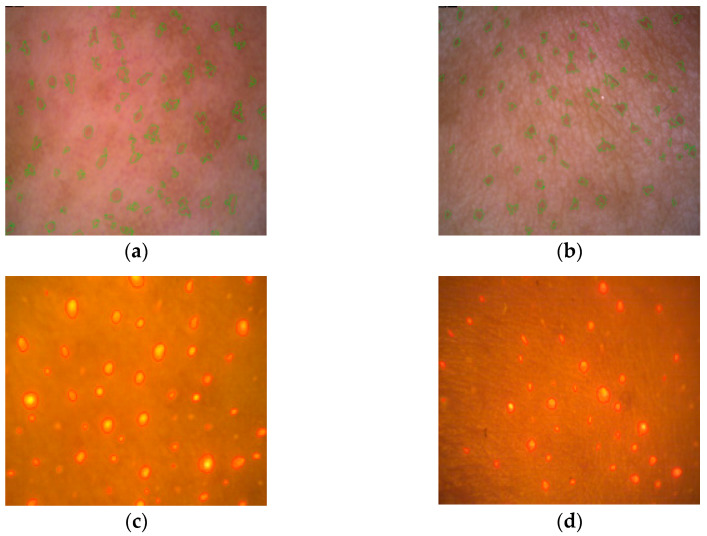
Examples of evaluating the number of pores from a scanned image of a volunteer’s skin in the T-zone, (**a**) baseline—7.0%, (**b**) after 28 weeks—5.4%; and porphyrin fluorescence at the chin site of the volunteer’s face, (**c**) baseline—6.1%, (**d**) after 28 weeks—2.9%.

A significant change (*p* < 0.05) in skin porosity is documented in Figure 2d. The differences between the measurements were most obvious on the forehead, nose, and chin, i.e., in the T-zone. Figure 3a,b show an example of evaluating the number of pores from a scanned image of the skin of a volunteer in the T-zone.

#### 2.3.4. Follicular Fluorescence

The fluorescence of porphyrins is documented in Figure 3c,d, where the orange-red spots indicate the presence of *C. acnes*. The number of bacteria monitored before and after therapy in relation to the area of the scanned part of the face corresponds to the image; see Table 2. Again, the T-zone (forehead, nose, and chin) turned out to be more problematic. The decrease in *C. acnes* in this area was up to 33%, and was 44% in the U-zone.

**Figure 4:**
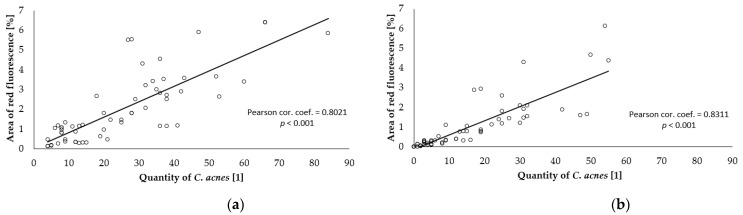
Correlation between the total area of red fluorescence and the amount of *C. acnes*: (**a**) baseline; (**b**) after 28 weeks.

**Figure 5:**
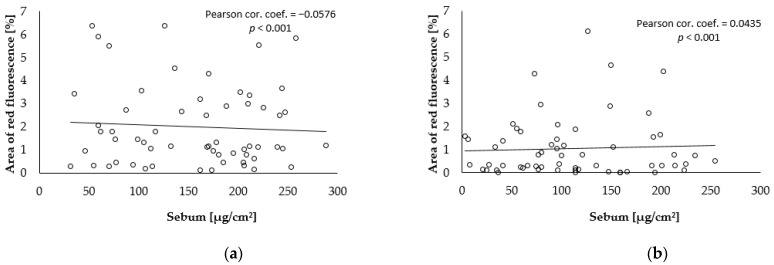
Correlation between the total area of red fluorescence and sebum: (**a**) baseline; (**b**) after 28 weeks.

**Figure 6:**
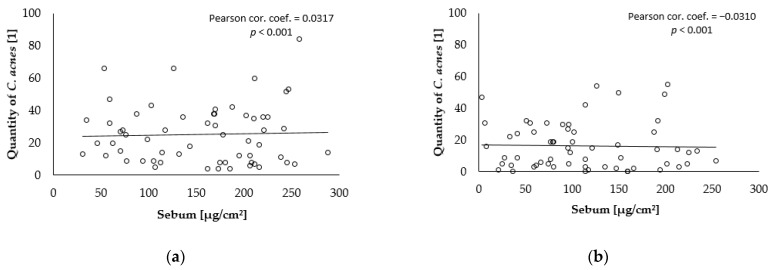
Correlation between the total area of the amount of *C. acnes* and sebum: (**a**) baseline; (**b**) after 28 weeks.

The degree of correlation was calculated between the values of parameters selected at the baseline level (before skin treatment) and after the end of the combined care treatment. The number of *C. acnes* was significantly positive in correlation with the porfyrin fluorescence scanned area, as presented in Figure 4. There was a significantly negative low correlation between the red fluorescence area and the total area of *C. acnes* on the amount of sebum; see Figure 5 and Figure 6.

## 3. Discussion

The study aimed to verify the effectiveness of combined care with a cosmetic product and laser therapy on a comedogenic skin type. The algorithm approach to acne treatment has no universally accepted recommended model [29]. Comedogenic skin is characterized mainly by mild noninflammatory lesions such as microcomedones and comedones, which are associated with a higher amount of sebum and a higher intensity of fluorescence compared to the late more severe inflammatory stages (pustules, papules, nodules and cysts). Objective visual assessment of initial microcomedonal lesions gradually turning into comedones is difficult. Its complexity is mentioned by study [16], which recommends porphyrin-induced fluorescence as not only a useful indicator of the presence of *C. acnes* but also for the evaluation of acne itself. According to different levels of sebum secretion, the face is commonly categorized into two zones, T and U; in the T-zone, sebum production is reported to be higher than in the U-zone. Publication [30] introduced the perioral zone, or the O-zone with medium to high sebum secretion, which is usually part of the T-zone, as another investigated zone with changes in sebum secretion, especially in men. The authors report higher levels of sebum not only on the face, but also on the neck, shoulders, and back, a lower level on the arms and legs, and the absence of sebum on the feet and palms. The level of sebum and the intensity of fluorescence expressed by the amount of *C. acnes* and the corresponding area in percentage were higher in the T-zone than in the U-zone on the skin of volunteers (see Table 2, Figure 1). This corresponds to the conclusions of the mentioned studies. This knowledge is practically usable in the process of formulating cosmetic products, which should be less greasy for application to these zones. It also indicates how important it is to correctly choose a product for the treatment of comedogenic areas of the face according to skin type, which can be different in different parts. 

In our study, it was found that there was a significant reduction in the sebum level during the 28-week application of the Lasocare Basic 645^®^ gel and its activation with the biostimulation laser, in both zones after 8 to 28 weeks from the start of monitoring the skin condition of the volunteers. The Lasocare^®^ method represents a combination of laser light and photosensitive enzymatic complexes with broad spectrum activity. The mechanism of action of *Lactoferrin* and *Lactoperoxidase* enzymes is described in a number of studies [31,32,33,34,35]. The effect of these enzymes on acne and skin inflammation has been studied not only in topically applied preparations but also in foods or dietary supplements containing them [33,36,37,38,39]. For example, two groups of patients who consumed *Lactoferrin*-enriched milk and nonenriched milk (placebo) for 12 weeks were tested for skin hydration, sebum, pH, and skin surface lipid content. The group consuming fortified milk showed an improvement in inflammation reduction, sebum reduction, and a reduction in the total number of lesions compared to the placebo group. Both groups showed a reduction in skin surface lipid levels, and the group that received *Lactoferrin* showed a reduction in triacylglycerol lipid content, which was correlated with a reduction in the number of acne lesions and the severity of acne. Hydration of the skin and pH did not change after supplementation [38]. *Lactoferrin*, as an optically active substance, has the ability to transmit light energy to the enzyme *Lactoperoxidase*, which catalyzes the oxidation of a number of inorganic and organic substrates. These oxidized intermediates have strong bactericidal effects, contributing to the inhibition of bacteria present on the skin of volunteers. The density of bacteria on the surface of the skin correlates with the intensity of fluorescence, and its decrease is directly related to the applied treatment, or in our case, to the applied care regimen. *C. acnes* synthesizes and releases specific porphyrins into the core of microcomedones, which participate in the fluorescence effect. The intensity of the fluorescence and the content of porphyrin are related and indicate the density of the *C. acnes* population in comedones. Therefore, the fluorescence intensity of individual pilosebaceous follicles is considered a marker of comedonal *C. acnes* colonization [40]. Lipid peroxidation in pilosebaceous ducts is believed to be responsible for the microaerophilic environment, which in turn contributes to further colonization of *C. acnes*. The authors of study [41] came to the interesting conclusion that red fluorescence may be related not only to the presence of *C. acnes* but probably also to sebum secretion, which was confirmed by a stronger correlation.

Fluorescence has been demonstrated in areas of increased pH, which would mean that substances formed as a result of higher pH also affect fluorescence. For normal skin, variable skin pH values have been reported in the literature [42,43], all in the acid range (“acid mantle“), with a wide range of pH from 4.0 to 6.0. The acidic surface pH and the pH gradient over the *stratum corneum* are important for optimal structure and function of the lipid barrier, cutaneous antimicrobial defense, and optimal epidermal differentiation. Maintaining an optimal pH value reduces the risk of skin reactions and the probability of damage caused by adverse external influences on the skin. Such influences may include excessive use of detergents and application of inappropriate cosmetic products. The pH increases in inflammatory skin conditions, facilitating microbial growth that leads to an increase in the *C. acnes* population and to the development of acne lesions. 

Another important physiological parameter that indicates the condition of the skin is the amount of water. The sebum present also contributes to the reduction of water loss, which, due to its composition, provides a protective function and is a natural skin emollient [44]. According to study [45], sufficiently hydrated skin with a good condition of the skin film produces a lower amount of sebum, and such a regulated skin barrier can be a suitable starting point for sebum reduction care. However, the gel was not formulated primarily as an occlusive agent and was not expected to significantly affect TEWL. Hydrating substances in the selected preparations for surface and deep skin cleansing and in the applied gel also contributed to maintaining a balanced water content in the *epidermis*. These substances included, e.g., *glycerin*, *propylene glycol*, *sodium lactate*, *sodium PCA*, *fructose*, *urea, mel* and others. In contrast, the slightly increased values of evaporation were probably caused by the treatment of the skin surface with a makeup remover before making the diagnosis. The makeup remover contained the surfactant *Ceteareth-25*, which could contribute to disrupting the protective natural skin lipid film and thus its permeability. According to [46], this imbalance may be associated with changes in the keratinization process; the authors also report that oily skin does not show statistically significant differences in hydration and water loss compared to normal skin, which were observed in sebum. On the other hand, the makeup remover contained apricot oil (*Prunus Armeniaca Kernel Oil*), which is very gentle on the skin and is capable of protecting the skin surface against dehydration and reduces clogged pores and excessive sebum production. It is suitable for treating problematic, acne-prone and oily skin [47,48]. The *Rosmarinus Officinalis Extract* contained in the composition of the makeup removal milk formulation and the chlorophyll complex together with *lactic acid* in the tonic had an anti-inflammatory effect on the skin. Their properties as a cleaning technique may have contributed to a decrease in inflammatory impacts in the areas monitored of the faces of the volunteers [49,50]. 

The data obtained showing a comparison of the skin condition of the volunteers before and after combined therapy indicate a reduction in the number of comedogenic lesions. The selected skin treatment products did not initiate a repeated new formation of comedones in the affected areas. The comedogenic potential of dermatological ingredients was documented as early as 1972 [51]. However, the comedogenic potential of the ingredients cannot be transferred to the entire product of which it is a part. On the other hand, it is not known whether new substances with a potential comedogenic effect arise due to interactions between ingredients during the production process. Therefore, it is important to clinically verify the final products using modern diagnostic technologies to ensure not only their effectiveness in controlled sebum secretion, but also their safety, as indicated in the study by Waranuch et al. [15]. 

The chosen multifactorial cosmetic care with gel laser therapy led to an effective reduction in the number of comedogenic lesions and proved to be one of the ways to prevent more serious forms of acne. The active ingredients contained in cosmetic skin cleansing products and the gel matrix with *Lactoferrin* and *Lactoperoxidase* enzymes, together with supporting therapeutic techniques, enhanced the synergistic effect according to the documented procedure with an evident improvement in skin condition within seven months. In comparison to emulsion-type matrices, the active ingredients contained in the gel matrix are more rapidly released. Enzymes that are more present rapidly inhibit the conversion of comedones into more severe acneiform impactions [52,53]. The clinical efficacy was comparable to that achieved with long-term topical or oral medications. The combined procedure is an alternative that offers a solution for sufferers for whom adherence to long-term regimens is a complication [54,55]. The condition of the skin significantly affects the physical attractiveness of an individual as well as their mental state. When communicating, it gives a first impression, despite its clear subjective perception. Problematic skin can lead not only to a negative perception of oneself but also of the society with which the individual is in daily contact.

## 4. Conclusions

Combined cosmetic gel treatment with laser therapy has demonstrably succeeded in reducing the number of comedogenic lesions on the face of volunteers. The use of instrumental noninvasive bioengineering methods led to a more precise diagnosis of the skin, its typing, and subsequently helped to choose the optimal procedure of professionally guided cosmetic care. By following these steps, excess sebum was removed while maintaining adequate hydration with minimal epidermal water loss. Controlling sebum secretion is key to preventing the development of more severe stages of acne. Professional cosmetic skin treatment with a cosmetic gel containing *Lactoperoxidase* and *Lactoferrin* (and without the presence of comedogenic ingredients) in combination with laser therapy, supplemented with regular home care, can be recommended as a suitable alternative to conventional forms of comedogenic skin treatment. The success of the chosen multifactorial care depends on its adherence and the participation of the person involved in the decision-making process. The advantage of such therapy is the relatively quick onset of the anticomedogenic effect, the support of skin regeneration, and maintenance of the skin’s optimal physiological function. Therefore, research in this area should lead to finding the most effective skin care methods that would help alleviate the early stages of acne and reduce recurrent conditions. 

## 5. Materials and Methods

### 5.1. Experimental Design

According to available publications, acne treatment consists of topically or orally applied medications, where their effects can be supported by laser treatment and a corresponding lifestyle. Among the literature studied, the following are presented as the baseline: (i) placebo values of different matrices without active agents [52,56]; (ii) baseline values of biophysical skin parameters before application of the studied formulations [57,58]; (iii) control values on half of the face without part of the therapy applied on the other half of the face [59]; or (iv) application of different therapies on each half of the face [60]. Based on these findings, an algorithm for the care of comedogenic skin type was designed, with the baseline condition of the volunteers’ skin after surface cleansing as the baseline. A placebo, e.g., a gel without active ingredients or a cosmetic therapy without gel and laser consisting only of superficial and deep cleansing of the skin would have insufficient or no effect achieved over a prolonged period with possible relapse states.

**Figure 7:**
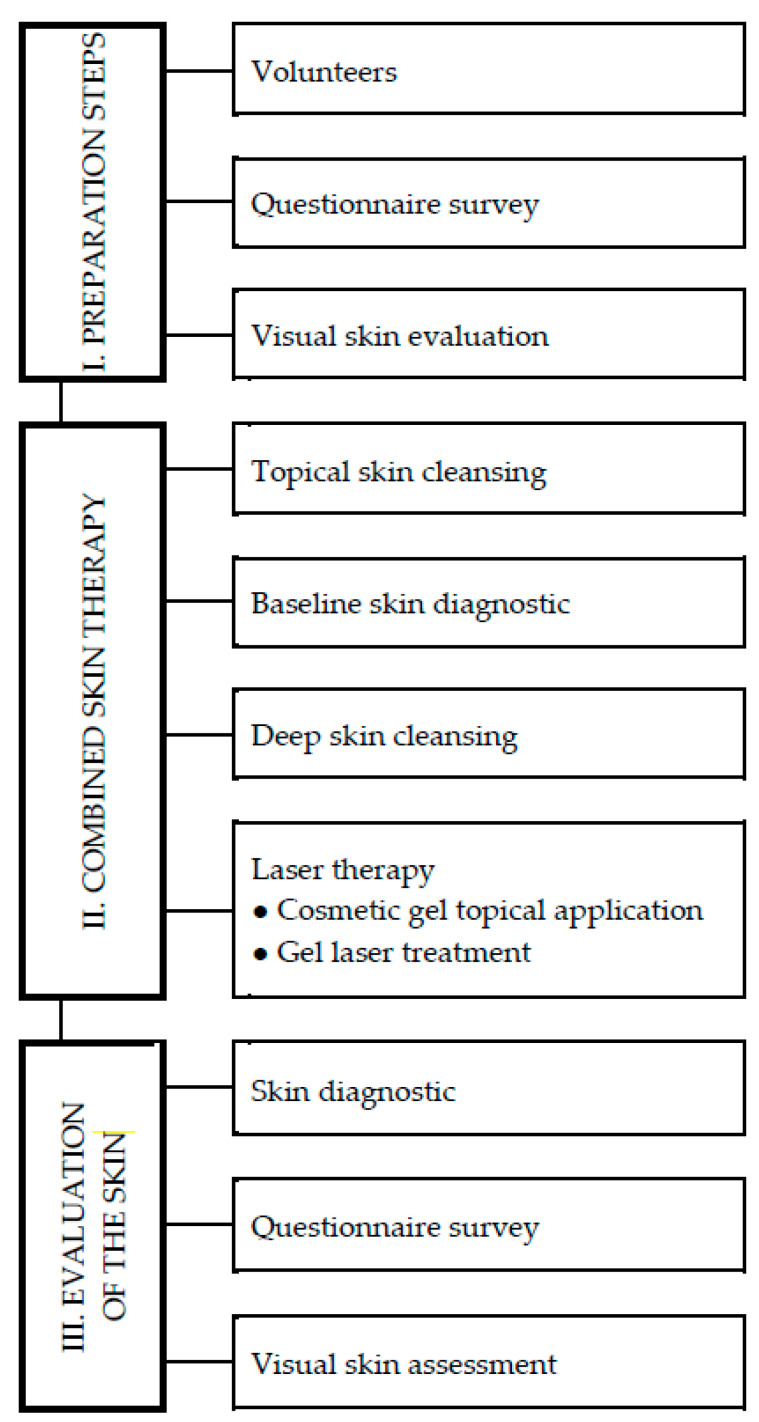
Organization of the experiment to verify the effects of combined cosmetic care and gel laser therapy on the parameters of comedogenic skin type.

Figure 7 shows a flow chart of the organization of the experiment verifying the effectiveness of the application of cosmetic gel and laser therapy to the parameters of the comedogenic skin type. The total duration of the experiment was 28 weeks, divided into 3 stages: (I) preparation steps consisting of the selection of volunteers, a questionnaire survey, and a visual evaluation of the volunteers’ skin; (II) combined skin therapy consisting of topical skin cleansing, baseline skin diagnostic, deep skin cleansing, and gel laser therapy; (III) evaluation of skin condition consisting of the skin diagnostic, questionnaire survey and visual evaluation of the volunteers’ skin.

### 5.2. Volunteers and Questionnaire Survey

Twelve women with comedogenic skin without other medical problems, 11–47 years old (average age 30 ± 13 years), were selected for the experiment. Volunteers were introduced to the purpose of the study, the procedure of treatment in the cosmetic studio, as well as to subsequent home treatment and the principles of diagnostic measurements. Before starting the experiment, the volunteers signed a health protocol and informed consent according to the International Ethical Guidelines for Biomedical Research Involving Human Subjects [61]. During the experiment, none of the volunteers visited a dermatologist or underwent any other type of cosmetic treatment. None of the volunteers used topical or oral medications that could affect the course of the experiment.

The questionnaire survey consisted of two parts. The first of them focused on determining the genetic predisposition of acne, the period of the first recorded problems, the forms and symptoms of comedogenic skin, the localization of comedones, and the mapping of the triggering factors that worsen the skin condition—diet, cosmetic products, stress, exposure to the sun, dehydration of the skin, menstruation, etc. The second part of the questionnaire aimed to obtain knowledge of the care and skin condition performed after the end of the experiment. 

### 5.3. Topical Skin Cleansing

Cosmetics for topical and deep cleansing meet the requirements for their safety and composition according to Regulation (EC) No. 1223/2009 on cosmetic products [62]. Topical cleansing was performed before the baseline skin diagnostics with a facial milk remover makeup (Syncare, Brno, Czech Republic) and subsequent cleansing with a facial moisturizing tonic (SynCare, Brno, Czech Republic) applied to a cleansing pad in circular motions. Composition of facial makeup removing milk: *Aqua*, *Octyldodecanol*, *Ethylhexyl Stearate*, *Cetereath-25*, *Prunus Armeniaca Kernel Oil*, *Cetyl Alcohol*, *Glyceryl Stearate*, *Glycerin*, *Tocopheryl Acetate*, *Rosmarinus Officinalis Extract*, *Phenoxyethanol*, *Ethylhexylglycerin*, *Citronellol*, *Linalool*, *Hydroxycitronellal*, *Geraniol*, *Parfum*, *Carbomer*, *Triethanolamine*. Composition of moisturizing tonic with chlorophyll: *Aqua*, *Glycerin*, *Propylene Glycol*, *Sodium Lactate*, *Sodium PCA*, *Glycine*, *Fructose*, *Urea*, *Niacinamide*, *Inositol*, *Sodium Benzoate*, *Lactic Acid*, *Cl 75810 (Chlorophyll complex)*, *Tocopheryl Acetate*, *Phenoxyethanol*, *Ethylhexyglycerin*, *Citral*, *Limonene*, *Citrus Medica*, *Limonum Oil.*

Deep cleansing of the skin was performed before skin therapy with a ten-minute application of a softening mask (Syncare, Brno, Czech Republic) composed of: *Aqua*, *Glycerin*, *Urea*, *Mel*, *Aloe Barbadensis Flower Extract*, *Citrus Limon Leaf Oil*, *Retinyl Palmitatem Dimethicone*, *Disodium EDTA*, *Propylene Glycol*, *Carbomer*, *Triethanolamine*, *Benzyl Alcohol*, *Ethylhexylglycerin*. 

### 5.4. Laser Therapy

At the beginning of laser therapy, a commercial Lasocare Basic 645^®^ cosmetic gel (Medistellar GmbH, Zug, Switzerland), meeting the requirements for its safety and composition according to Regulation (EC) No. 1223/2009 on cosmetic products [62], was applied and massaged on the face of the volunteers. Composition of the gel: *Aqua*, *Propylene Glycol*, *Sorbitol*, *C12-15 Pareth-12*, *Xanthan Gum*, *PEG/PPG-20/20*, *Dimethicone*, *Phenoxyethanol*, *Glycerin*, *Sodium Dehydroacetate*, *Sodium Phytate*, *Lactoperoxidase*, *Glucose Oxidase*, *Biotin*, *Tocopherol*, *Potassium Phosphate*, *Glucose Pentaacetate*, *Lactoferrin*, *Potassium Thiocyanate*, *Disodium Phosphate*, *Tromethamine*, *Alcohol*, *Ethylhexylglycerin*, *Pentaerythrityl*, *Tetra-di-t-butyl*, *Hydroxyhydrocinnamate*, *Methylisothiazolinone.*

Then, a 12 min laser procedure was applied to activate the enzyme complex *Lactoferrin* and *Lactoperoxidase* contained in the cosmetic gel. Laser therapy was performed with a panoramatic scanner consisting of 12 mutually independent emitters with a power of 5 mW and a wavelength of 645 nm assembled in an arc (Medistellar GmbH, Zug, Switzerland). Then, for the entire 28 weeks of skin therapy, the gel was applied repeatedly at 24 h intervals every evening at home. After 4 and 8 weeks of therapy, the 12 min laser procedure was repeated. Professional cosmetic and laser treatment, including diagnostics, was performed in an air conditioned room at a temperature of 23 to 25 °C and a relative humidity of 40 to 45%.

### 5.5. Skin Diagnostic

#### 5.5.1. Visual Skin Assessment

**Figure 8:**
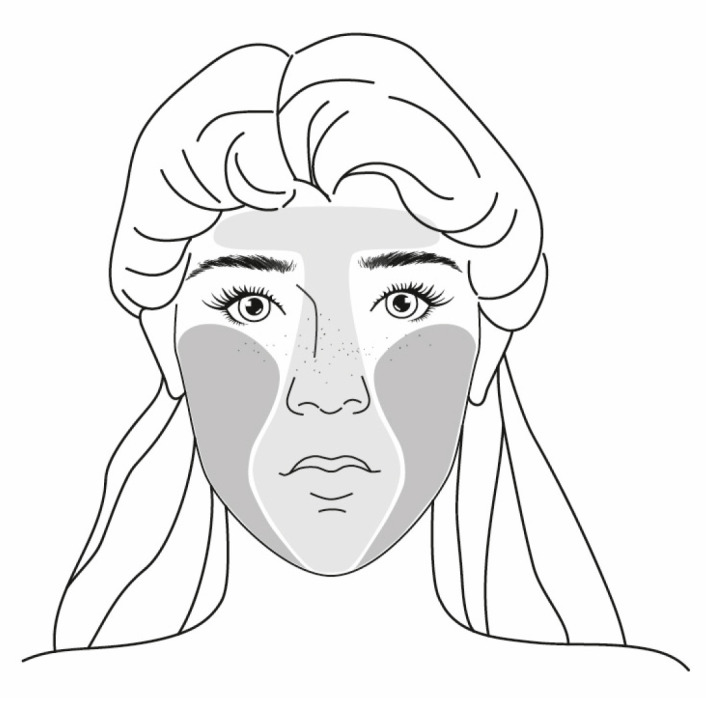
Areas of measurement and clinical evaluation of the face of volunteers.

Comedonic severity was assessed using a three-level score: score 1—presence of comedones, score 2—presence of comedones and papules, score 3—presence of comedones, papules and pustules. Skin evaluation was performed before and after therapy in five parts of the face. The results are presented as the T-zone (high sebum secreting zone) including the forehead (midglabella), nose (nose tip), chin (mental prominence), and the U-zone (low sebum secreting zone), including the right and left cheeks (the most prominent areas), and as a whole (T + U zone); see Figure 8.

#### 5.5.2. Noninvasive Instrumental Bioengineering Methods

The objective characterization of the skin condition was diagnosed by a series of noninvasive bioengineering methods. To compare the results of combined cosmetic gel and laser therapy treatment with blank treatment, a baseline diagnostic was performed after topical skin cleansing. The evaluation of the skin condition was performed after 4, 8 and 28 weeks of combined therapy.

The platform for diagnosing skin sebum, hydration, TEWL, and skin pH was the MPA 5 station (Courage and Khazaka Electronic GmbH, Cologne, Germany). The level of sebum was detected photometrically with Teflon tape of Sebumeter^®^ SM 815 in μg/cm^2^. Sebum was collected from each zone of the face (see Figure 8) on a plastic strip using a constant pressure of 10 N for 30 s. For individual zones (T- and U-zone) and the whole face (T + U-zone), the amount of sebum was expressed using Equations (1–3) given in the publications [13,63]. The detected amount of sebum was recorded and classified according to Table 3.

(1) T-zone=∑Sebum of forehead,nose,chin/3

(2) U-zone=∑Sebum of right and left cheeks/2

(3) T+Uzone =∑Sebum of forehead,nose,chin,right and left cheeks/5

The state of skin hydration was assessed with a Corneometer^®^ CM 825 probe, which detects the water content in the *stratum corneum* based on measurements of electrical capacity. Skin hydration was measured five times in each part of the face, and the average value from these measurements was expressed as the result for T-zone, U-zone and T + U zone. The results were expressed in corneometry units (c.u.) according to the level of hydration indicated in the device manual, where the values <30 c.u. indicate extremely dry skin, the values of 30–40 c.u. indicate dry skin, and the values >40 c.u. indicate normal skin [65]. TEWL was measured with a Tewameter^®^ TM 300 probe, which determines the pressure gradient of water vapor according to Fick’s law, to evaluate the integrity of the skin barrier function, and it is expressed in g/h.m^2^. Monitoring of water loss was performed in each part of the face 15 times; the first 5 values were neglected due to equalization of temperature and humidity in the probe chamber and the skin of the volunteers. An average value was calculated for each part of the face for a given zone. The starting point for interpreting the results was a scale that characterizes the condition of the skin in a range of 0–10 g/h.m^2^ for very healthy condition, 10–15 g/h.m^2^ for healthy condition, 15–25 g/h.m^2^ for normal condition, 25–30 g/h.m^2^ strained skin and above 30 g/h.m^2^ for critical condition [66]. The acidity of the skin mantle was measured with a Skin-pH-Meter^®^ PH 905 probe. This specially designed probe consists of a flat-topped glass electrode for full skin contact, connected to a voltmeter. The system measures potential changes due to the activity of hydrogen cations, the changes in voltage are displayed as pH, which was interpreted according to [67] in range: less than 3.5–4.3 (acidic range), 4.5–5.5 (normal), and more than 5.7 (high) skin pH value. The measurement was performed in each area of the face once, and then, the average value for the T-zone, U-zone, T + U zone was calculated.

The number of pores in the skin was scanned by a Visioscope^®^ PC35 camera (Courage and Khazaka Electronic GmbH, Cologne, Germany) and using Complete Skin Investigation evaluation software (Courage and Khazaka Electronic GmbH, Cologne, Germany), a percentage calculation was performed based on the area of the scanned image [68]. 

The degree of follicular fluorescence was captured by a Visiopor^®^ PP34N camera (Courage and Khazaka Electronic GmbH, Cologne, Germany). The camera uses a specific ultraviolet A (UVA) light with a wavelength of 375 nm, in which the porphyrins become visible as fluorescent orange-red spots in the pores, indicating the presence of *C. acnes* bacteria inhabiting the comedones. The parameters analyzed by the software were the number and percentage of the 4.0 × 5.5 mm area covered with orange-red spots [69]. The analysis of the number of pores and the degree of follicular fluorescence in individual areas of the face was expressed as an average for the T, U and T + U zone. 

### 5.6. Statistical Analysis

All statistical analyses of biophysical characteristics were conducted using Microsoft Office Excel (version 10, Microsoft, Santa Rosa, California, CA, USA). Data are expressed in terms of the average ± standard deviation and were calculated for the T-zone, U-zone and T + U zone. Differences between baseline parameters of and other measurements were statistically evaluated with a paired *t* test; *p* < 0.05 was considered statistically significant. Pearson’s correlation was used to investigate possible associations between fluorescence quantity, lesion counts, and sebum levels in the five facial areas at baseline and fourth measurement.

## Figures and Tables

**Table 1:** Results of the visual evaluation of the volunteers (score 1–3) before and after lasotherapy.

Zones	Distribution of Volunteers according to Comedonic Severity		
	Score 1 Comedones	Score 2 Comedones and Papules	Score 3 Comedones, Papules and Pustules
	Baseline/28 Weeks	Baseline/28 Weeks	Baseline/28 Weeks
T + U zone	3/1 2/5 0/0	3/1 2/4 0/0	1/0 0/0 1/1
T-zone			
U-zone			

**Table 2:** Red fluorescence expressed by the amount of *C. acnes* and the area before and after therapy.

Red Fluorescence				
Time	Baseline		28 Weeks	
Zone	Quantity of *C. acnes*	Area (%)	Quantity of *C. acnes*	Area (%)
T + U zone	25.2 ± 2.3	2.0 ± 0.2	16.3 ± 1.9 *	1.1 ± 0.4 *
T-zone	31.9 ± 3.0	2.6 ± 0.3	21.5 ± 2.7 *	1.4 ± 0.2 *
U-zone	15.2 ± 2.7	1.2 ± 0.3	8.5 ± 1.8 *	0.6 ± 0.2 *

* statistically significant difference from baseline (*p* < 0.05).

**Table 3:** Classification of objective facial skin type by reference values of sebum levels measured with the Sebumeter^®^ [64].

Skin Type	Sebum Levels [μg/cm^2^]		
	T + U Zone (Whole Face)	T-Zone	U-Zone
Dry	<88	<100	<70
Normal	88–204	100–220	70–180
Oily	˃204	˃220	˃180

## Data Availability

Data sharing not applicable.
